# Fluid restriction management in the treatment of COVID-19: a single-center observational study

**DOI:** 10.1038/s41598-022-22389-5

**Published:** 2022-10-15

**Authors:** Yosuke Matsumura, Takuya Sugiyama, Natsuki Kondo, Masaya Miyahara, Noriyuki Hanaoka, Hideaki Nagashima, Yuki Kasahara, Naohiko Fujiyoshi, Azusa Inada, Shin Inaba

**Affiliations:** 1 Department of Intensive Care, Chiba Emergency Medical Center, 3-32-1, Isobe, Mihama, Chiba, Chiba 261-0012 Japan; 2 Department of Anesthesiology, Chiba Emergency Medical Center, Chiba, Chiba, Japan

**Keywords:** Viral infection, Infectious diseases

## Abstract

The relationship between fluid management and the severity of illness, duration of treatment, and outcome of coronavirus disease 2019 (COVID-19) is not fully understood. This study aimed to evaluate whether weight change during hospitalization was associated with COVID-19 severity, length of hospital stay, and route of admission. In this study, we assessed the effectiveness of fluid restriction management in patients with severe COVID-19. COVID-19 patients admitted to our hospital between July 2020 and October 2021 were analyzed. Patients were treated with standard drug therapy based on the Japanese guidelines and respiratory support according to the severity of the disease. Early enteral nutrition, defecation management, and anticoagulation therapy were also administered. Fluid restriction management was performed using furosemide and continuous renal replacement therapy as needed unless hemodynamic instability or hyperlactatemia was present. Patient background, route of admission (ambulance, A; transfer, T), weight at admission and discharge, the severity of illness (oxygen therapy, G1; mechanical ventilation, G2; extracorporeal membrane oxygenation, G3), in-hospital mortality, and length of hospital stay were analyzed. There were 116 subjects: G1 (n = 48), G2 (n = 43), and G3 (n = 25), with ages (median [IQR]) of 58 (47–70), 65 (53–71.5), 56 (51–62) years, 40 (83.3%), 31 (72.1%), and 19 (76.0%) males, respectively. Hospital stays were 4.5 (2–7), 10 (7–16), and 18 (15–26) days, and the in-hospital mortality rates were 0 (0%), 7 (16.3%), and 8 (32%), respectively. Body mass index on admission was 26 (23.1–30.2), 27.1 (22.7–31.1), and 31.5 (27.1–33.1) kg/m^2^, and weight loss during admission was 1.1 (0–2.9), 4.6 (2.3–5.7), 9.2 (5.6–10.5) kg (P < 0.001, Jonckheere–Terpstra test. Weight loss in the severe group (G2 + G3) was 3.4 (0.5–5.8) kg [A, n = 12] and 5.6 (4.4–9) kg [T, n = 43] [P = 0.026, Mann–Whitney U test]. The lengths of hospital stay were 5 (2–7), 9 (7–15), and 18 (12–26) days [P < 0.001, Jonckheere–Terpstra test]. In our fluid restriction management, patients with severe COVID-19 had significant longer hospital length of stay, weight loss, especially those who were transferred to the hospital.

## Introduction

Some patients with coronavirus disease 2019 (COVID-19) become severely ill and require intensive care. During a pandemic, preventing severe disease and shortening the duration of treatment are desirable for individual patients and are necessary to maintain the health care system.

Among the treatment modalities for COVID-19, pharmacotherapy, such as antiviral drugs^1–4^, neutralizing antibodies^5^, immunosuppressive/modulating drugs^6–8^, respiratory supportive therapy such as high-flow nasal cannula (HFNC)^9^, ventilator, extracorporeal membrane oxygenation (ECMO)^10^, prone positioning^11^, and anticoagulation^12^ have been reported and evaluated. In non-COVID-19 ARDS and sepsis, fluid restriction management has been associated with improved oxygenation and treatment duration^13–16^.

However, there are no reports on whether fluid management in COVID-19 is associated with the severity of the illness, duration of treatment, and outcome. It is unclear whether fluid restriction management contributes to the prevention of deterioration and improvement of oxygenation, thereby shortening the length of the hospital stay.

We hypothesized that the severity of COVID-19 was related to duration of treatment and weight loss. Disease severity was classified based on the presence or absence of mechanical ventilation or ECMO. Weight change during hospitalization was recorded. We examined the relationship between illness severity and weight loss, hospital length of stay (LOS), and in-hospital mortality. We then compared the weight loss by admission route.

## Methods

### Patients

COVID-19 patients admitted to our hospital between July 2020 and October 2021 were included in the analysis. The study period corresponds to the second to fifth waves in Japan. We mainly treated patients with severe respiratory failure (requiring ventilatory support) or those with the potential for severe respiratory failure and patients who were COVID-19 positive and require emergency care (surgery, endovascular therapy, etc.) [“COVID-19 positive emergency patients”]. We often collaborate with the hospitals in charge of mild and moderate illnesses to accommodate patients transferred to the hospital. In some cases, patients with severe respiratory failure are brought in directly by ambulance, and difficult-to-accommodate cases are accepted during nights and holidays to maintain the emergency medical system.

### The institutional policy of COVID-19 treatment

In principle, the COVID-19 treatment policy was based on the Coronavirus Disease 2019 (COVID-19) Treatment Guidelines^17^. The Japanese guidelines are publicly available and have been updated. The essential drug therapy was remdesivir and dexamethasone (6 mg/day)^6^. The steroid (methylprednisolone) dose was increased to 2 mg/kg when the patient was judged to be critically ill based on CT, oxygenation, and rate of deterioration. Patients without bacterial infection or immunosuppression were treated with tocilizumab or baricitinib, in addition to remdesivir and steroids. Until the third wave, HFNC was only used after extubation. After the fourth wave (May 2021), HFNC was actively introduced before mechanical ventilation^9^. If excessive inspiratory effort and tachypnea are not resolved even after HFNC is started, tracheal intubation and mechanical ventilation should be started as soon as possible. In the patients with severe obesity^18^, young age, no delirium, and severe chronic obstructive pulmonary disease, HFNC with high oxygen concentration could be continued with awake prone patients^11^ to avoid artificial respiration. In mechanically ventilated patients, esophageal pressure was monitored to titrate the positive end-expiratory pressure (PEEP)^19^. When the CT showed a dorsal or unilateral predominant shadow distribution, positional therapy (prone or lateral position) was used. Although indications of the ECMO are very different, such as refractory hypoxia or hypercapnia, VV-ECMO was introduced.

Patients on ventilators or ECMO were administered continuous enteral nutrition starting at 20 mL/h with peptide-based formula (Peptamen AF^®^, Nestle HealthCare Nutrition, Inc.) and increased the injection rate daily. The target calorie dose (approximately 1500 kcal/day) is usually achieved on day 4. In addition to continuous intravenous insulin, oral hypoglycemic agents (biguanides, dipeptidyl peptidase-4 inhibitors, sodium-glucose cotransporter-2 inhibitors, and thiazolidinediones) have been used to control glucose intolerance due to steroid use and frequent complications of diabetes mellitus. To control excessive inspiratory effort, patients with severe COVID-19 often require high doses of opioids. Naldemedine^20^ was used in addition to magnesium oxide and sodium picosulfate to promote intestinal peristalsis. Since most severe COVID-19 cases were hemodynamically stable, we attempted fluid restriction management. We restricted the injection fluid and used diuretics or continuous renal replacement therapy if needed in acute kidney injury or chronic kidney disease patients.

### Measurement

The patients’ background (age, sex, length, weight, blood test on admission) and admission route (direct ambulance transport, A; transfer from the outside hospital, T) were recorded. Severity was defined as oxygen therapy (non-intubated, Group 1 [G1]; ventilated, Group 2 [G2]; ECMO, Group 3 [G3]). We collected data on ventilator duration, ECMO duration, drug treatment (total steroid dose, tocilizumab, baricitinib), sequelae, blood transfusion requirement, in-hospital mortality, hospital length of stay (LOS), and waiting period (days between extubation and transfer or discharge).

Patient characteristics, clinical course, and weight loss during hospitalization were compared according to illness severity. In the severe group (G2 + G3). We then compared the characteristics, clinical course, and admission route. We examined the hospital LOS and waiting period for each severity of illness among the patients who were discharged alive.

### Statistical analyses

Scale data are expressed as median (25th–75th percentile) and categorical data as number (percentage). Group comparisons were made using the Jonckheere–Terpstra test or Mann–Whitney U test for scale data and Chi-square test for categorical data, with P < 0.05 considered significant. The primary outcome was hospital LOS, and the secondary outcomes were in-hospital mortality and the waiting period.

### Ethics approval

This study was approved at the institutional review board at Chiba Emergency Medical Centre (Oct 11, 2021). This research has been performed in accordance with the Declaration of Helsinki and relevant guidelines/regulations. Informed consent was waived by the Ethics Committee.

## Results

A total of 116 patients were included in the analysis: G1 (n = 48), G2 (n = 43), and G3 (n = 25), with ages (median [25th–75th percentile]) of 58 (47–70), 65 (53–71.5), and 56 (51–62) years, respectively; 40 (83.3%), 31 (72.1%), and 19 (76.0%) were male. COVID-19 positive emergency patients were multiple trauma [fasciotomy and cervical spine posterior fixation], middle finger amputation [re-adhesion], and stroke. They were categorized in G1, and did not require special fluid management. Body weight at admission was 76.4 (66.3–90.5) kg, 70 (59–82.6) kg, 83.4 (73.2–100) kg, and BMI was 26 (23.1–30.2), 27.1 (22.7–31.1), 31.5 (27.1–33.1) kg/m^2^. White blood cell counts were 6.3 (4.9–8.95), 7.4 (4.7–10.7), 8.5 (5.8–12.7)/10^3^ μL, and lymphocyte fractions were 12.6 (8.6–20), 8.3 (4.4–13.7), 9.3 (5.7–13.2) %. Ferritin was 762.3 (392.1–1400.2), 1156.2 (607.6–1526.7), 1052.7 (598.7–1666.8) ng/mL and d-dimer was 2.1 (1.8–2.9), 2.7 (2.1–3.9), 3 (2.3–6.3) μg/mL (Table 1).

**Table 1:** Baseline characteristics of the studied patients on admission.

	Group 1 (N = 48)	Group 2 (N = 43)	Group 3 (N = 25)
Age, years	58 (47–70)	65 (53–71.5)	56 (51–62)
Male, n (%)	40 (83.3)	31 (72.1)	19 (76.0)
Transfer, n (%)	26 (54.2)	33 (76.7)	20 (80)
Height, cm	171 (163–178)	167 (160–170)	166 (160–171)
Weight, kg	76.4 (66.25–90.5)	70 (59–82.6)	83.4 (73.2–100)
BMI, kg/m^2^	26 (23.1–30.2)	27.1 (22.7–31.1)	31.5 (27.1–33.1)
Lactate, mmol/L	1.5 (1.15–1.85)	1.45 (1.2–1.95)	1.5 (1.1–1.9)
WBC, /10^3^μL	6.3 (4.9–8.95)	7.4 (4.7–10.7)	8.5 (5.8–12.7)
Hb, g/dL	14.4 (13–15.3)	13.4 (12.75–14.4)	14.1 (12.9–14.9)
Ht, %	41.4 (38.3–44.2)	39.5 (36.8–42.1)	40.8 (37.9–42.6)
Plt, /10^3^μL	199 (168–249)	217 (154–253)	208 (171–257)
Neu, %	82.1 (74.2–87.4)	87.5 (83.1–91.6)	87.7 (82.5–91.5)
Lym, %	12.6 (8.6–20)	8.3 (4.4–13.7)	9.3 (5.7–13.2)
Mo, %	4.5 (3.1–6.2)	2.9 (2.2–5)	3 (2–5.2)
Eo, %	0.1 (0–0.3)	0 (0–0)	0 (0–0)
Ba, %	0.1 (0.1–0.2)	0.1 (0.1–0.2)	0.2 (0.1–0.2)
Glucose, mg/dL	142 (117–164.5)	179 (144.5–220.5)	181 (131–209)
TP, g/dL	6.5 (6–6.9)	6.2 (5.9–6.7)	6.2 (5.9–6.7)
Albumin, g/dL	3.2 (2.9–3.6)	2.8 (2.7–2.9)	2.9 (2.6–3.2)
BUN, mg/dL	16 (13–20)	20 (17–31)	22 (15–29)
Creatinine, mg/dL	0.8 (0.7–0.95)	0.8 (0.6–1.1)	0.8 (0.6–1.3)
T-bil, mg/dL	0.6 (0.5–0.8)	0.6 (0.45–0.8)	0.7 (0.4–1.2)
CRP, mg/dL	4.39 (2.5–11.75)	7.99 (3.45–15.6)	13.09 (4.64–17.85)
CK, /UL	99 (57–163)	103 (53–241)	334 (102–452)
HbA1c, %	6.5 (6–7.7)	6.6 (6.1–7.6)	6.7 (6.4–7.3)
Mg, mg/dL	2.2 (2–2.3)	2.2 (2–2.35)	2.2 (1.9–2.3)
IP, mg/dL	3 (2.6–3.5)	3.6 (3–4.35)	3.4 (2.6–4.2)
Ferritin, ng/mL	762.3 (392.1–1400.2)	1156.2 (607.6–1526.7)	1052.7 (598.7–1666.8)
PT-INR	1.02 (0.97–1.18)	1.06 (1.02–1.14)	1.06 (1.01–1.13)
APTT, s	30.8 (28.9–34.7)	34.8 (30.1–44.1)	33 (28.7–44.5)
Fibrinogen, mg/dL	543 (468–606)	579 (478–678)	602 (504–686)
d-Dimer, μg/mL	2.1 (1.8–2.9)	2.7 (2.1–3.9)	3 (2.3–6.3)

*BMI *body mass index, *WBC* white blood cell, *Hb* hemoglobin, *Ht* hematocrit, *Plt* platelet, *Neu* neutrophil, *Lym* lymphocyte, *Mo* monocyte, *Eo* eosinophil, *Ba* basophil, *TP* total protein, *BUN* blood urea nitrogen, *T-bil* total bilirubin, *CRP* C-reactive protein, *CK* creatinine kinase, *Mg* magnesium, *IP* inorganic phosphorus, *PT-INR *prothrombin time-international normalized ratio, *APTT* activated partial thromboplastin time.

HFNC was performed in 23 (47.9%, G1) of the non-ventilated patients. HFNC was attempted prior to mechanical ventilation in 12 (27.9%, G2), and 11 (44%, G3) patients. The ventilator duration was 6 (4–9) days in G2 and 14 (10–17.5) days in G3, and the ECMO duration was 8 (6–11) days. The total dose of dexamethasone–equivalent steroids during the hospital stay was 31 (3.5–46), 54 (40–109), and 152 (59–559) mg. Tracheostomy was performed in five (11.6%) and five (20%) patients in G2 and G3, respectively, and chest drainage was performed in seven (16.3%) and four (16%) patients, respectively. Heparin-induced thrombocytopenia (HIT) was observed in 4 (9.3%) and 5 (20%) patients, respectively. The lowest platelet count during hospitalization was 189 (151–248), 155 (108–245), 86 (52–158) 10^3^/μL, the highest ferritin level was 686.4 (392–1273.9), 1032.6 (526.8–1683.2), 1002.9 (576.5–1515.6) ng/mL, the highest d-dimer level was 2.6 (2–4), 4.6 (3.1–15), 18.7 (8.1–26) μg/mL. The hospital LOS was 4.5 (2–7), 10 (7–16), and 18 (15–26) days, and in–hospital mortality rates were 0 (0%), 7 (16.3%), and 8 (32%), respectively (Table 2).

**Table 2:** Clinical course of the studied patients.

	Group 1 (N = 48)	Group 2 (N = 43)	Group 3 (N = 25)
HFNC, n (%)	23 (47.9)	12 (27.9)	11 (44)
MV duration, days		6 (4–9)	14 (10–17.5)
ECMO duration, days			8 (6–11)
Total dexamethasone, mg	31 (3.5–46)	54 (40–109)	152 (59–559)
Tocilizumab, n (%)	7 (14.6)	13 (30.2)	8 (32)
Baricitinib, n (%)	1 (2.1)	4 (9.3)	5 (20)
Tracheostomy, n (%)	0 (0)	5 (11.6)	5 (20)
Chest drainage, n (%)	0 (0)	7 (16.3)	4 (16)
HIT, n (%)	0 (0)	4 (9.3)	5 (20)
Packed red blood cell, mL			560 (280–1400)
Fresh frozen plasma, mL			2400 (720–2880)
Platelet, mL			725 (450–800)
Platelet, lowest, 10^3^/μL	189 (151–248)	155 (108–245)	86 (52–158)
Highest Ferritin, ng/mL	686.4 (392–1273.9)	1032.6 (526.8–1683.2)	1002.9 (576.5–1515.6)
Highest d-dimer, μg/mL	2.6 (2–4)	4.6 (3.1–15)	18.7 (8.1–26)
Hospital LOS, days	4.5 (2–7)	10 (7–16)	18 (15–26)
In-hospital mortality, n (%)	0 (0)	7 (16.3)	8 (32)

*HFNC* high flow nasal cannula, *MV* mechanical ventilation, *ECMO* extracorporeal membrane oxygenation, *HIT* heparin-induced thrombocytopenia, *LOS* length of stay.

**Figure 1:**
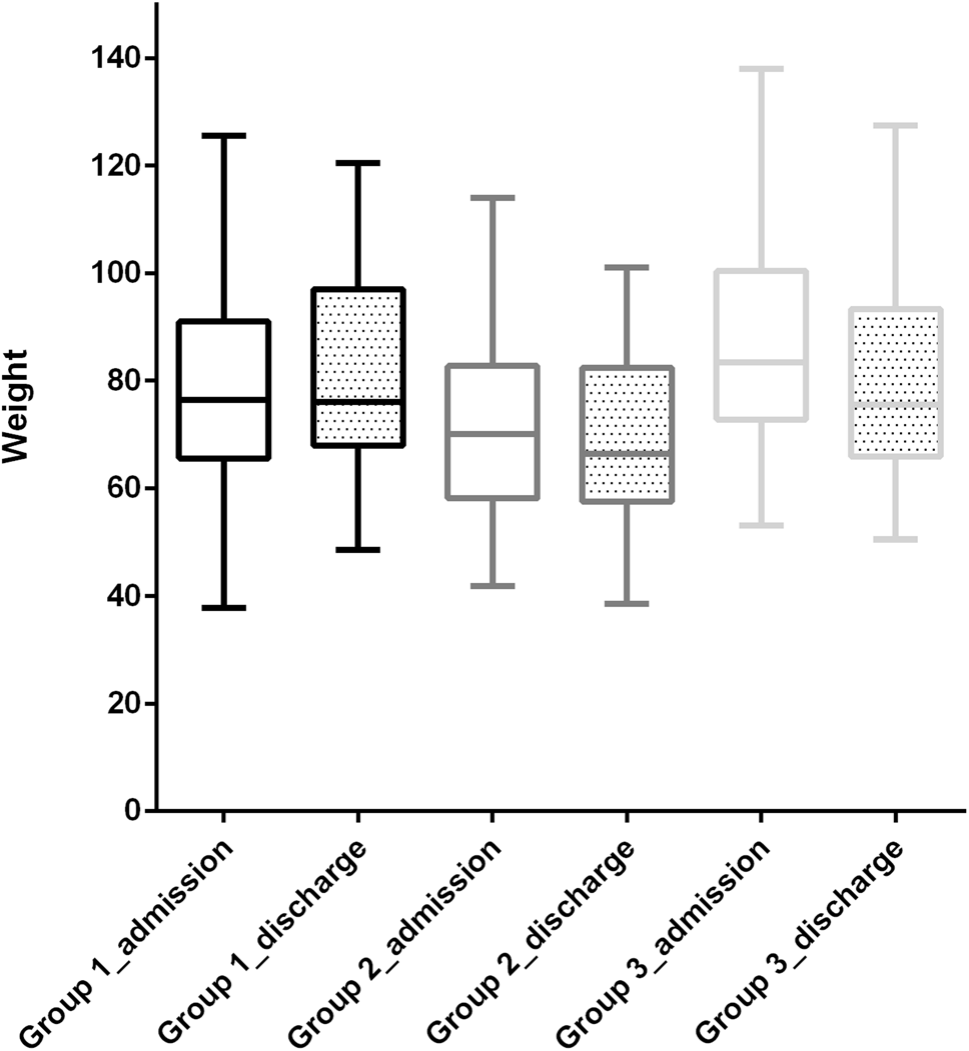
Comparison of body weight on admission and discharge. Weights on admission in Group 1, Group 2, and Group 3 were 76.4 (66.25–90.5), 70 (59–82.6), 83.4 (73.2–100) kg, respectively. Weights at discharge were 76.0 (67.9–97.0), 66.3 (57.4–82.4), 93.3 (65.9–93.3) kg, respectively.

Weight loss during the hospitalization was 1.1 (0–2.9), 4.6 (2.3–5.7), and 9.2 (5.6–10.5) kg [P < 0.001, Jonckheere–Terpstra test] (Fig. 1, Table 3). The weight loss by admission route was 3.4 (0.5–5.8) kg for ambulance transport patients (Ambulance, A) (N = 12) and 5.6 (4.4–9) kg for transfer patients (Transfer, T) [P = 0.026, Mann–Whitney U test]. There were no differences in patient background, sequelae, hospital LOS, or in-hospital mortality according to route (Table 4). The ventilator duration in survivors was 5 (4–7) days in G2 (n = 36) and 12 (9–14) days in G3 (n = 17) [P < 0.001, Mann–Whitney U test], and the ECMO duration in survivors was 7 (6–10) days. The hospital LOS was 5 (2–7), 9 (7–15), and 18 (12–26) days, respectively [P < 0.001, Jonckheere–Terpstra test], and the waiting days were 2 (1–3.5) and 3 (1–7) days for G2 and G3, respectively (Table 5).

**Table 3:** Weight loss during the admission.

	Group 1 (N = 48)	Group 2 (N = 43)	Group 3 (N = 25)	P value
Weight on admission, kg	76.4 (66.3–90.5)	70 (59–82.6)	83.4 (73.2–100)	
Weight on discharge, kg	76 (68.5–94.2)	66.3 (57.9–82.4)	75.5 (66.7–91.5)	
Weight loss, kg	1.1 (0–2.9)	4.6 (2.3–5.7)	9.2 (5.6–10.5)	< 0.001

Jonckheere–Terpstra Test.

**Table 4:** Comparison of characteristics according to admission route.

	Ambulance (N = 12)	Transfer (N = 43)	P value
Age, years	68 (53–71)	59 (51–68)	0.43
HFNC, n (%)	6 (40)	17 (32.1)	0.56
HIT, n (%)	2 (13.3)	7 (13.2)	1
Tracheostomy, n (%)	1 (6.7)	9 (17)	0.44
Chest drainage, n (%)	2 (13.3)	9 (17)	1
Weight loss, kg	3.4 (0.5–5.8)	5.6 (4.4–9)	0.026
MV duration, days	7 (5–11)	9 (5–14)	0.68
ECMO duration, days	8 (7–8)	9 (6–12)	0.72
Total dexamethadone, mg	74 (50–167)	66 (40–192)	0.62
Hospital LOS, days	12 (7–17)	15 (8–24)	0.36
In-hospital mortality, n (%)	3 (20)	12 (22.6)	1

Mann–Whitney U test or Chi-square test.

*HFNC* high flow nasal cannula, *HIT* heparin-induced thrombocytopenia, *MV* mechanical ventilation, *ECMO* extracorporeal membrane oxygenation, *LOS* length of stay.

**Table 5:** Hospital length of stay and waiting period for transfer in the survived patients.

	Group 1 (N = 48)	Group 2 (N = 36)	Group 3 (N = 17)	P value
MV duration		5 (4–7)	12 (9–14)	< 0.001
ECMO duration			7 (6–10)	N/A
Hospital LOS	4.5 (2–7)	9 (7–15)	18 (12–26)	< 0.001
Waiting days		2 (1–3.5)	3 (1–7)	0.33

Mann–Whitney U test.

*MV* mechanical ventilation, *ECMO* extracorporeal membrane oxygenation, *LOS* length of stay.

## Discussion

More significant weight loss was observed in the severe group (G2 + G3). The higher the severity of the illness, the greater the weight loss and more extended hospitalization. The longer hospitalization may be related to a higher chance of malnutrition due to intestinal malabsorption and atrophy of the intestinal mucosa. Patients transported directly by ambulance experienced less weight loss than those transferred to the hospital. As the severity of the disease increased, the hospital LOS increased, but the waiting period was short in both G2 and G3.

Age and sex were similar across all severity levels. A male preponderance was a common trend (Table 1)^21^. The ECMO group (G3) had a heavier body weight and BMI than the ventilator group (G2) and the non-intubated group (G1). Although esophageal pressure monitoring was performed to confirm transpulmonary pressure^19^, the high-pressure setting in a large patient may have been a factor in introducing ECMO. Decreased lymphocyte counts^22^ and increased ferritin levels^23^ reported in patients with severe COVID-19 were also observed in the admission findings of this study (Table 1). The D-dimer level at admission was only mildly elevated in each group; however, the maximum d-dimer level during the admission period was higher in the severe group^24^. It may be related to the onset of HIT and ECMO. Tracheotomy and chest drainage were required in some cases in the severe group. The causes of the high frequency of pneumothorax and mediastinal emphysema may include a combination of factors, including the pathogenesis of COVID-19, steroids, and positive pressure ventilation^25^. Although HFNC therapy was rarely used in the early stages of the pandemic due to concerns about aerosol generation, it later became the primary means of oxygen therapy. Twelve patients (27.9%) of Group 2 and 11 (44%) of Group 3 received HFNC therapy before intubation. This data may suggest that HFNC is an oxygen therapy worth trying before intubation; however, it is not uncommon for patients to deteriorate and require ventilation or ECMO, so it is preferable to perform it in an environment that allows for a switch to intensive care.

Since patients with severe COVID-19 rarely have circulatory failure, we attempted to manage fluid restriction or active fluid removal from the time of admission to the ICU, unless there was hemodynamic instability or hyperlactatemia. More significant weight loss in the severe respiratory failure group could be influenced by malnutrition due to extended hospitalization despite the proactive enteral nutrition strategy (Table 3). The cause of respiratory failure in COVID-19 could be hyperpermeability and microcirculatory disturbance due to hypercytokinemia^26^; however, excessive fluid in the lungs may also be a factor. Previous observational studies have also suggested the effect of furosemide in COVID-19 patients^27,28^. A systematic review suggested the benefit of deresuscitation, defined as active fluid removal in the critically ill or injured patients^29^.

In a comparison by route of admission, patients who were transported by ambulance (A) had less weight loss than those who were transferred from an outside hospital (T) (Table 4). When the number of patients exceeded the capacity of hospitalization (January and August 2021), even those requiring hospitalization were forced to stay at home. Many patients transported to the emergency room may have been dehydrated upon admission. However, patients transferred to the hospital were already receiving fluids, and some may have been overhydrated.

Due to the isolated nature of hospitalization, fluid balance and weight trends in patients with mild-to-moderate COVID-19 are rarely controlled. Excess fluid during the treatment of moderately ill patients may contribute to disease severity. To date, there is no solid evidence regarding the efficacy of fluid restriction therapy for COVID-19. We aim to learn more about COVID-19 while adapting fluid management to other intensive care fields^30^.

HFNC was used in almost half of the G1 cases. Many G2 patients discontinued HFNC and started ventilation; therefore, it is unlikely that unnecessary patients were ventilated. The ventilator duration in the ventilator group (G2) was 5 (4–7) days, which was shorter than the estimated duration from previous analyses (7.97 [5.29–11.18] days)^31^. Fluid restriction management may have contributed to the short duration of ventilator use in this study. The length of hospital stay for ventilated patients (G2) was 9 (7–15) days and the waiting period was 2 (1–3.5) days, while for ECMO patients (G3), it was 18 (12–26) days and 3 (1–7) days.

This study has several limitations. First, owing to the nature of observational studies, no causal relationship can be concluded from this study. Second, due to the single-center study design, patient demographics and bias in treatment strategy (HFNC, ventilator, ECMO threshold, and choice of immunosuppressive drugs) may not be incorporated into other institutions. Third, the overall sample size and subgroups (ambulance/transfer) were small, and a third of the patients were not ventilated. Despite these limitations, the severity of COVID-19 was related to hospital length of stay and weight loss.

## Conclusions

Weight loss during hospitalization was more significant in patients with severe COVID-19; it was more remarkable, especially in patients transferred to the hospital than in those transported directly by ambulance. The duration of the ventilator and hospital LOS increased with the severity of the disease but was shorter than previously estimated.
